# Evaluating selection bias in a population-based cohort study with low baseline participation: the LIFE-Adult-Study

**DOI:** 10.1186/s12874-019-0779-8

**Published:** 2019-07-01

**Authors:** Cornelia Enzenbach, Barbara Wicklein, Kerstin Wirkner, Markus Loeffler

**Affiliations:** 1 Institute for Medical Informatics, Statistics, and Epidemiology, University of Leipzig, Haertelstrasse 16-18, 04107 Leipzig, Germany; 2 LIFE - Leipzig Research Centre for Civilization Diseases, University of Leipzig, Philipp-Rosenthal-Strasse 27, 04103 Leipzig, Germany

**Keywords:** Participation, Selection bias, Validity, Reasons for nonparticipation, Cohort study

## Abstract

**Background:**

Participation in epidemiologic studies is steadily declining, which may result in selection bias. It is therefore an ongoing challenge to clarify the determinants of participation to judge possible selection effects and to derive measures to minimise that bias. We evaluated the potential for selection bias in a recent population-based cohort study with low baseline participation and investigated reasons for nonparticipation.

**Methods:**

LIFE-Adult is a cohort study in the general population of the city of Leipzig (Germany) designed to gain insights into the distribution and development of civilisation diseases. Nine thousand one hundred forty-five participants aged 40–79 years were randomly sampled in 2011–2014. We compared LIFE-Adult participants with both the Leipzig population and nonparticipants using official statistics and short questionnaire data. We applied descriptive statistics and logistic regression analysis to evaluate the determinants of study participation.

**Results:**

Thirty-one percent of the invited persons participated in the LIFE-Adult baseline examination. Study participants were less often elderly women and more often married, highly educated, employed, and current nonsmokers compared to both the Leipzig population and nonparticipants. They further reported better health than nonparticipants. The observed differences were considerable in education and health variables. They were generally stronger in men than in women. For example, in male study participants aged 50–69, the frequency of high education was 1.5 times that of the general population, and the frequency of myocardial infarction was half that of nonparticipants. Lack of time and interest, as well as health problems were the main reasons for nonparticipation.

**Conclusions:**

Our investigation suggests that the low baseline participation in LIFE-Adult is associated with the typical selection of study participants with higher social status and healthier lifestyle, and additionally less disease. Notably, education and health status seem to be crucial selection factors. Consequently, frequencies of major health conditions in the general population will likely be underestimated. A differential selection related to sex might also distort effect estimates. The extent of the assessment, the interest in the research topic, and health problems of potential participants should in future be considered in LIFE-Adult and in similar studies to raise participation and to minimise selection bias.

**Electronic supplementary material:**

The online version of this article (10.1186/s12874-019-0779-8) contains supplementary material, which is available to authorized users.

## Background

Participation has declined over the past decades for all types of epidemiologic studies [1]. The decreased willingness to participate in an epidemiologic study may threaten the validity of the results. Those who volunteer for study participation are often more likely to have favourable exposure and health profiles compared to those who do not. Consequently, estimates of prevalence, incidence, and exposure-disease associations may be biased. This error is referred to as response bias or, more broadly, selection bias [2]. Although being a potentially important precondition for the validity of an epidemiologic study, participation is often insufficiently reported in the publication of the results [1, 3].

The presence of selection bias can usually not be inferred from the study data alone. We need to compare study participants with nonparticipants or with the target population in terms of relevant characteristics to judge possible selection effects on the study results [4, 5]. For such comparisons, we have to collect some core information from nonparticipants as well, using short questionnaires or secondary data. In addition, data on the target population may be obtained from official statistics or representative surveys.

Using these methods, the potential for selection bias has been investigated in epidemiologic studies in the general population for many years (e.g., [6–15]. These studies have predominantly shown that participants in baseline examinations of cohort studies and in cross-sectional studies are more likely to be female and to have higher social status, healthier lifestyles, and better subjective health than nonparticipants. Results are contradictory with respect to age and prevalent diseases. These observations have been made for participation rates of mainly above 50%.

The LIFE-Adult-Study is a recent population-based cohort study conducted in the city of Leipzig, Germany [16]. An extensive programme consisting of questionings, physical examinations, and biologic specimen collections was established to better understand the distribution and the development of civilisation diseases. With a response of about 30%, the participation in LIFE-Adult was markedly lower than in previous cohort and cross-sectional studies that had examined selection bias. In light of this low participation and the claimed higher susceptibility of studies with low levels of participation to selection bias [1, 17], we were seeking for an in-depth understanding of the determinants of response in our study.

Our primary objective was to evaluate the potential for selection bias in LIFE-Adult applying two independent methods: (1) the comparison of LIFE-Adult participants with the Leipzig population with regard to socio-demographic and lifestyle characteristics using official statistics and (2) the comparison of LIFE-Adult participants with nonparticipants additionally considering health-related variables by means of short questionnaire data. Furthermore, we investigated reasons for nonparticipation given in the short questionnaire by describing their distribution and their relations to the individuals’ characteristics.

## Methods

### Study design and participants

#### LIFE-Adult-Study

LIFE-Adult is a cohort study designed (1) to estimate prevalences and incidences of common diseases and subclinical phenotypes in the adult population of Leipzig and (2) to investigate the interplay of molecular-genetic and lifestyle factors in the development of these conditions.

Participants in LIFE-Adult are an age and gender stratified random sample of the general population of Leipzig mainly aged 40 to 79 years, which was drawn by the registration offices. All selected residents were sent an invitation letter with information on the study. Persons who had not responded within four weeks received a reminder letter. Those who had not responded within further two weeks were contacted by phone (see reference [16] for more details on recruitment).

The baseline assessment took place between August 2011 and November 2014. All participants underwent a core assessment consisting of interviews and questionnaires, physical examinations, and collection of blood and urine (average duration 5 to 6 h). Participants aged 60 to 79 years were invited to additional assessments focusing on cognitive function and depressive symptoms on two further days (average duration 3 to 4 h each).

The assessments were conducted in the LIFE-Adult study centre, which is located in the city centre and easy to reach. Participants received 20 Euro per visit to cover their travel expenses. They were also offered selected examination results in written form. In addition, several public relation activities were organised to raise participation.

Persons unwilling to participate in LIFE-Adult were asked to fill in a short questionnaire, which was enclosed in the first invitation and the reminder letter since January 2012. The questionnaire comprised 17 questions related to socio-demography, lifestyle, health status, and reason for nonparticipation.

In the present investigation, we included participants in LIFE-Adult who were in the study’s main age range from 40 to 79. For the comparison with short questionnaire participants by means of regression analysis, we further restricted the population to study participants who had received the first invitation since January 2012. Out of all short questionnaire participants, we considered those aged between 40 and 79.

#### Census and microcensus

We obtained data on the Leipzig population from the census and the microcensus.

Data on the sex and age distribution within Leipzig come from the national census, which is conducted every ten years [18]. The data represent population updates by 30 June 2013 (based on census data from May 2011). At that time, about half of the LIFE-Adult population was recruited.

The microcensus is a representative statistics of the population and labour market conducted annually in Germany [19]. The sample comprises 1 % of all households. A fixed set of socio-demographic characteristics is assessed each year using mainly computer-assisted interviews in the households. Respondents are obligated to answer these questions, resulting in high response figures (e.g., unit-response 97.6% and item-response > 97% in the year 2013 [19]). Additionally, variable topics are addressed every four years on a voluntary basis. We used public microcensus data of the year 2013 representing the annual average. For each characteristic, extrapolated numbers per sex and age strata were available. To prevent misinterpretation due to random error, numbers less than 7000 for a given strata are generally not released and numbers below 10,000 should be interpreted cautiously. We had to consider this when selecting and handling the analysis variables.

### Variables

We selected major risk factors and health conditions as variables for analysis. For the comparison of LIFE-Adult participants with the Leipzig population, we considered sex and age, as well as marital status, education, employment, and smoking status. For the comparison of LIFE-Adult participants with short questionnaire participants, we additionally chose physical condition and medically diagnosed myocardial infarction, stroke, diabetes, and cancer. We did not consider those items of the short questionnaire for which corresponding data were not available from study participants (e.g., sports activities) or for which the assessment methods were not comparable between the two populations (weight status).

A detailed definition of each analysis variable in each population is given in Additional file 1: Table S1.

### Data analysis

#### Calculation of participation

**Fig. 1:**
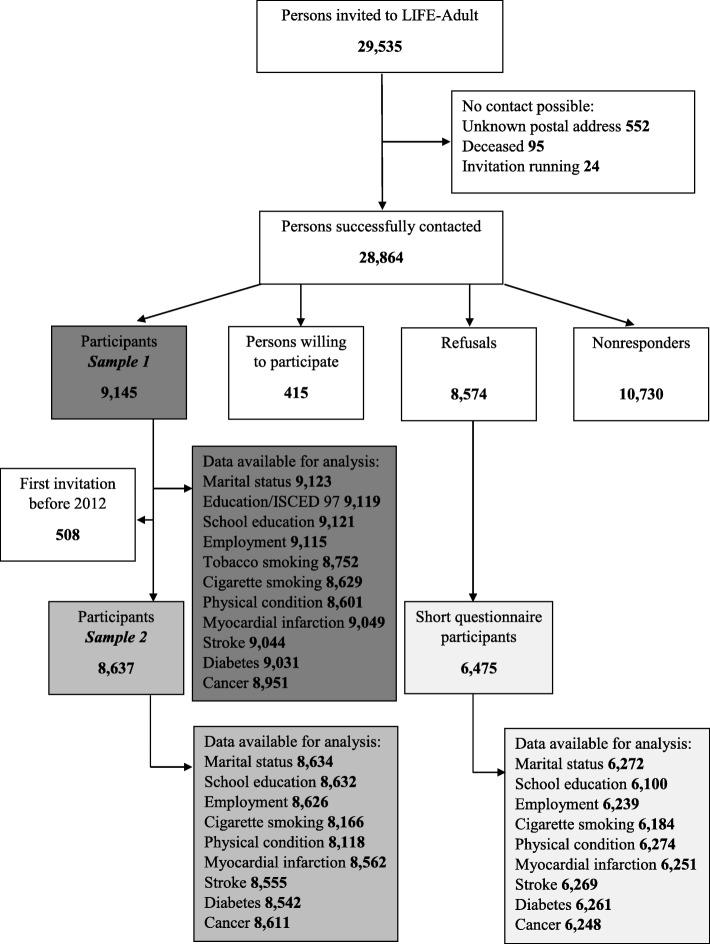
Participation in LIFE-Adult and in the short questioning, age range 40 to 79 years. *Sample 1* of LIFE-Adult participants was used for the comparison with the Leipzig population and with short questionnaire participants using descriptive statistics (see Table 1). *Sample 2* of LIFE-Adult participants was used for a more detailed comparison with short questionnaire participants using logistic regression (see Table 2, as well as the “Methods” section for further explanation). *Invitation running* refers to those invitees who had been sent an invitation few weeks before the end of the recruitment and who did not respond within that time frame. *Persons willing to participate* are those invitees who had agreed to participate in LIFE-Adult but did not get an appointment because the targeted total number of participants had been achieved. *Refusals* are those invitees who actively declined to participate by means of a response form enclosed in the invitation letters or by phone. *Nonresponders* are those invitees who entirely ignored the invitation. *Data available for analysis* refers to the number of non-missing data for each variable. Missing data include questioning and item nonresponse, the answer categories “I don’t know” and “refusal of answer”, and erroneous data. ISCED 97 = International Standard Classification of Education 1997

We calculated participation in LIFE-Adult using two different measures. The *response proportion* is the percentage of persons that participated out of the total number of persons who had been eligible for study [20]. Our denominator comprised LIFE-Adult participants, persons willing to participate, refusals, nonresponders, and persons who could not be contacted, including persons with unknown address, those who had died before contact could be made, and persons with running invitations (see Fig. 1 for illustration and explanation of the categories of individuals). We also calculated the *recruitment efficacy proportion* by excluding from the denominator those nonparticipant categories that cannot be influenced much by the investigator [21], namely the persons who could not be contacted and those willing to participate.

We calculated participation in the short questioning by relating the number of short questionnaire participants to all invited persons who did not participate, namely refusals, nonresponders, persons who could not be contacted, and persons willing to participate.

#### Comparison of LIFE-Adult participants with the Leipzig population and short questionnaire participants

We compared LIFE-Adult participants with the Leipzig population and with short questionnaire participants using descriptive statistics. We thereby investigated whether there were sex or age differences in selective participation. For this, we calculated relative frequencies of study variable values according to sex and 10-year age groups. We dichotomised variable values and chose reference groups in a way that ensured reliable microcensus data. As only summary data were available from official statistics, we could not indicate the precision of the estimated frequencies at this stage of analysis.

We investigated the differences between LIFE-Adult and short questionnaire participants in more detail by means of logistic regression, taking into account the uncertainty of the estimates and explanatory factors. We estimated odds ratios and 95% confidence limits. Participation in LIFE-Adult was the dependent variable. In a first model series, we included each analysis variable separately as independent variable. In a second model series, we analysed the association of each variable with study participation controlling for differences in the age distribution between study and short questionnaire participants. In a third model series, we examined to what extent the observed associations may be attributed to differences in social status by additionally including school education as independent variable. We estimated all associations separately for men and women according to the observations in the descriptive analysis.

#### Calculation of completeness of the data

For all analysis variables, we calculated the completeness of the data for LIFE-Adult and short questionnaire participants by sex and age. Completeness is defined as the number of non-missing data divided by the total number of the population. Missing data include questioning and item nonresponse, the answer categories “I don’t know” and “refusal of answer”, and erroneous data.

#### Analysis of reasons for nonparticipation

The reason for nonparticipation had been asked in the short questionnaire by the question “For which reasons do you not want to participate in our study? Please state the most important reason.” The answer categories comprised lack of time, job-related reasons, no interest, doubts about the value of the study, health reasons, moved, language reasons, no information on reasons, other reason: which one.

Before the analysis, we combined non-exclusive categories, namely “lack of time” and “job-related reasons”, “no interest” and “doubts about the value of the study”, and “no information on reasons” and missing data. If possible, we matched answers in the category “other reason” to more meaningful categories. However, we subsumed categories with very few cases (moved and language reasons) in the category “other reason”. We checked the “comment” field for nonparticipation reasons and replaced missing data if possible. We further checked the fields “other reason” and “comment” to possibly identify the most important reason in case of multiple answers.

We calculated relative frequencies of the final reasons for nonparticipation for all respondents and according to sex, age (40 to 64 vs. 65 to 79 years), and school education as an indicator of social status.

We used SPSS (IBM SPSS Statistics), version 24, for our calculations.

## Results

### Participation in LIFE-Adult and in the short questioning

The numbers of individuals aged 40 to 79 at different stages of the study are presented in Fig. 1. Nine thousand one hundred forty-five persons participated in LIFE-Adult, resulting in a response proportion of 31% and a recruitment efficacy proportion of 32.1%. Among nonparticipants, 6475 persons filled in the short questionnaire, corresponding to a participation rate of 31.8%.

### Participants in LIFE-Adult in comparison with the Leipzig population and short questionnaire participants

**Fig. 2:**
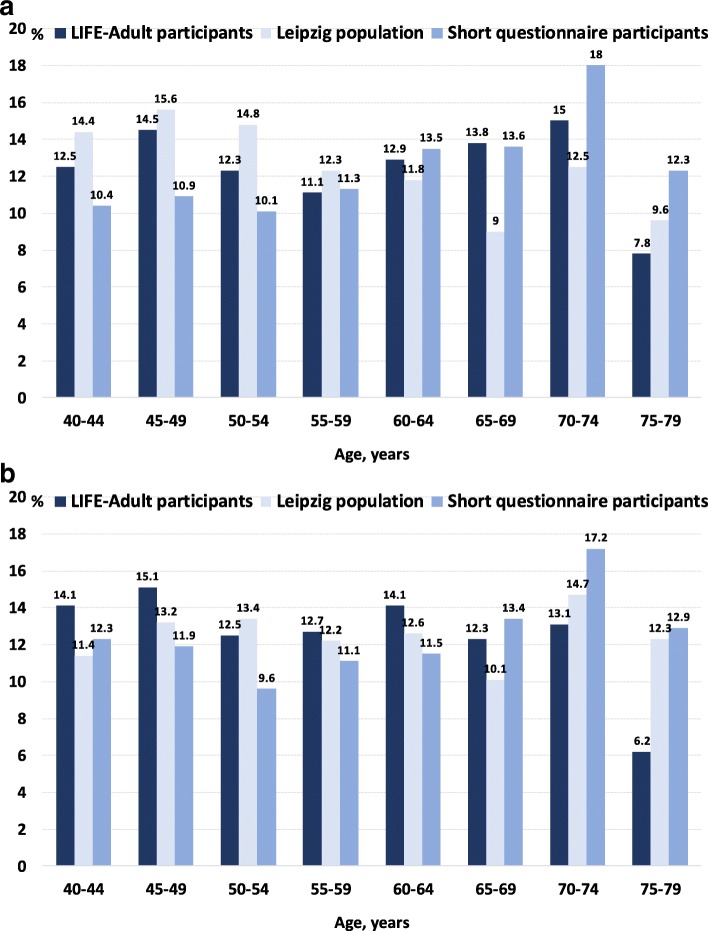
Age distribution in LIFE-Adult participants, the Leipzig population, and short questionnaire participants. **a.** Men **b.** Women

In comparison with the Leipzig population, the percentage of women aged 75 to 79 was considerably lower in LIFE-Adult (6.2% vs. 12.3%, Fig. 2). Compared to short questionnaire participants, the percentage of both women and men aged 75 to 79 was markedly lower in LIFE-Adult (women: 6.2% vs. 12.9%, men: 7.8% vs. 12.3%).

LIFE-Adult participants differed from the Leipzig population in all other selected characteristics (Table 1). They were more often married at ages 40 to 69 years in both sexes. They were higher educated in all age groups with stronger differences observed in men. They were more often employed in the considered age from 40 to 59 in both men and women. Finally, they were more often current nonsmokers in men. The differences between LIFE-Adult participants and the Leipzig population were most pronounced in school education. For example, the frequency of 1st stage tertiary education in male study participants was 1.5 times the frequency in the male Leipzig population in the age range 50 to 69 (see Table 1 for the corresponding frequencies). Regarding the other variables, the frequencies in LIFE-Adult were less than 1.2 times the frequencies in the Leipzig population.

**Table 1:** Characteristics of LIFE-Adult participants, the Leipzig population, and short questionnaire participants by sex and age

	Men				Women			
Age, years	40 to 49	50 to 59	60 to 69	70 to 79	40 to 49	50 to 59	60 to 69	70 to 79
Married, %								
LIFE-Adult participants	48.8	65.3	80.7	85.3	52.7	65.8	68.1	55.5
Leipzig population	44.7	60.5	69.7	84.6	49.2	59.2	60.2	57.5
SQ participants	48.2	60.2	73.5	81.9	56.3	60.5	66.0	55.9
Highly educated, %								
LIFE-Adult participants								
1st stage tertiary education	46.6	50.6	62.1	73.2	51.0	51.5	47.7	43.7
Hochschulreife^a^	34.7	32.6	34.8	34.8	34.7	31.4	27.4	21.1
Leipzig population								
1st stage tertiary education	35.4	34.6	(40.2)	56.0	45.5	41.1	35.3	36.8
SQ participants								
Hochschulreife^a^	32.4	24.8	27.1	27.8	33.2	26.9	25.2	16.5
Employed, %								
LIFE-Adult participants	90.7	84.0	31.3	4.2	90.9	83.9	26.1	2.2
Leipzig population	86.2	79.6	(38.9)	/	84.6	75.7	(24.9)	/
SQ participants	85.2	76.6	23.9	2.9	83.3	75.4	17.2	1.9
Current nonsmoker, %								
LIFE-Adult participants								
Nonsmokers of tobacco	66.1	68.7	82.2	93.0	71.1	71.4	87.0	94.6
Nonsmokers of cigarettes	67.4	70.0	83.9	94.4	71.4	71.7	87.2	94.6
Leipzig population								
Nonsmokers of tobacco	57.7	63.1	73.6	92.1	70.0	74.7	85.0	97.0
SQ participants								
Nonsmokers of cigarettes	59.8	62.3	75.8	87.5	67.3	67.1	86.0	92.9
Poor physical condition, %								
LIFE-Adult participants	2.2	3.7	4.6	4.6	3.0	4.2	5.0	6.0
SQ participants	2.7	5.3	6.6	11.7	3.0	3.6	4.9	9.9
Myocardial infarction, %								
LIFE-Adult participants	0.5	2.5	5.2	9.2	0.1	0.9	1.8	2.6
SQ participants	0.7	4.9	10.0	13.1	0.7	1.1	2.5	4.4
Stroke, %								
LIFE-Adult participants	0.7	2.1	3.4	5.9	0.4	1.9	2.2	3.4
SQ participants	0.8	2.3	5.8	9.3	0.2	2.3	3.3	5.1
Diabetes, %								
LIFE-Adult participants	3.4	9.7	20.5	23.0	1.8	6.3	13.4	19.8
SQ participants	6.0	13.2	30.1	34.7	3.5	12.7	21.0	28.1
Cancer, %								
LIFE-Adult participants	2.2	4.6	12.1	24.0	6.7	8.2	13.9	18.9
SQ participants	2.9	7.1	10.8	21.5	4.8	10.1	14.7	18.0

Data for the Leipzig population: Percentages corresponding to less than 7000 cases are marked by “/”, percentages corresponding to less than 10,000 cases are given in parenthesis. ^a^Hochschulreife = technical college or university entrance qualification, SQ = short questionnaire

When comparing LIFE-Adult with short questionnaire participants, similar and additional differences were observed (Table 1). LIFE-Adult participants were more often married among those older than 50 years, particularly in men. They had a higher school qualification and were more often current nonsmokers in all ages with greater differences in men. LIFE-Adult participants were more often employed in all age groups and in both sexes. They were less often in poor physical condition among men in all ages but particularly at the age of 70 to 79. In women, this difference was observed only in the oldest age group. LIFE-Adult participants reported less often to have been diagnosed with myocardial infarction and diabetes, irrespective of age and sex. With regard to stroke, there was an analogous difference among those older than 60 years. As to the frequency of diagnosed cancer, inconsistent and generally small differences between the two populations were found across age and sex strata. The deviations of LIFE-Adult from short questionnaire participants were particularly pronounced in education and health variables. For example, the frequency of high education in male study participants was 1.3 times that of male short questionnaire participants in the age range 50 to 69. For myocardial infarction, the corresponding ratio was 0.5. Including in the analysis only those LIFE-Adult participants invited since the beginning of the short questioning did not affect the aforementioned differences (data not shown).

In the logistic regression analysis, in both sexes the odds of being participant in LIFE-Adult was lower among those aged 70 to 79, having low or no school qualification, being in poor physical condition, and having been diagnosed with myocardial infarction, diabetes, or stroke, whereas it was higher among those being employed (Table 2, model 1). In addition, in men, the odds of being LIFE-Adult participant was lower among current smokers, whereas in women it was higher among former smokers.

**Table 2:** Associations of individuals’ characteristics with study participation: LIFE-Adult participants versus short questionnaire participants

	Men			Women		
	Model 1	Model 2	Model 3	Model 1	Model 2	Model 3
Aged 40 to 44 y	Reference			Reference		
Aged 45 to 49 y	1.11 (0.92–1.35)			1.14 (0.96–1.36)		
Aged 50 to 54 y	0.99 (0.81–1.21)			1.16 (0.97–1.39)		
Aged 55 to 59 y	0.81 (0.67–0.99)			1.01 (0.84–1.21)		
Aged 60 to 64 y	0.80 (0.66–0.97)			1.07 (0.90–1.28)		
Aged 65 to 69 y	0.85 (0.70–1.03)			0.80 (0.67–0.96)		
Aged 70 to 74 y	0.69 (0.58–0.83)			0.68 (0.58–0.81)		
Aged 75 to 79 y	0.53 (0.43–0.65)			0.42 (0.35–0.51)		
POS/Realschule^a^	Reference	Reference		Reference	Reference	
Hochschulreife^a^	1.13 (1.01–1.26)	1.16 (1.04–1.29)		1.06 (0.95–1.18)	1.07 (0.96–1.19)	
Hauptschule^a^	0.40 (0.35–0.47)	0.45 (0.38–0.53)		0.45 (0.39–0.52)	0.56 (0.48–0.66)	
Other/no qualification	0.56 (0.42–0.76)	0.58 (0.42–0.78)		0.33 (0.24–0.45)	0.35 (0.25–0.48)	
Married	1.08 (0.98–1.20)	1.25 (1.12–1.39)	1.20 (1.08–1.34)	1.04 (0.95–1.14)	1.02 (0.93–1.12)	1.00 (0.91–1.10)
Employed	1.61 (1.47–1.78)	1.56 (1.36–1.78)	1.42 (1.24–1.64)	1.79 (1.64–1.96)	1.63 (1.43–1.86)	1.53 (1.34–1.75)
Never smoker^b^	Reference	Reference	Reference	Reference	Reference	Reference
Former smoker^b^	0.90 (0.80–1.00)	0.94 (0.84–1.05)	0.98 (0.87–1.10)	1.33 (1.18–1.50)	1.19 (1.05–1.34)	1.19 (1.05–1.34)
Current smoker^b^	0.72 (0.63–0.81)	0.62 (0.54–0.70)	0.69 (0.60–0.79)	1.07 (0.95–1.20)	0.86 (0.76–0.98)	0.88 (0.77–1.00)
Poor physical condition	0.50 (0.40–0.62)	0.55 (0.44–0.69)	0.60 (0.48–0.75)	0.79 (0.64–0.97)	0.91 (0.73–1.12)	0.99 (0.79–1.22)
Myocardial infarction	0.50 (0.40–0.61)	0.56 (0.46–0.70)	0.57 (0.46–0.70)	0.50 (0.36–0.72)	0.61 (0.43–0.87)	0.65 (0.46–0.94)
Stroke	0.58 (0.46–0.75)	0.67 (0.52–0.86)	0.68 (0.53–0.88)	0.61 (0.45–0.83)	0.71 (0.52–0.96)	0.79 (0.58–1.09)
Diabetes	0.55 (0.48–0.62)	0.60 (0.52–0.68)	0.62 (0.54–0.71)	0.50 (0.44–0.58)	0.58 (0.51–0.67)	0.62 (0.53–0.71)
Cancer	0.87 (0.75–1.01)	1.03 (0.88–1.20)	1.01 (0.86–1.19)	0.91 (0.79–1.04)	1.02 (0.88–1.17)	1.02 (0.88–1.18)

Association measures are odds ratios (95% confidence limits). The dependent variable is participation in LIFE-Adult vs. participation in the short questioning. Model 1: crude association of each analysis variable with study participation, model 2: adjustment for age (40 to 44, 45 to 54, 55 to 64, 65 to 69, 70 to 74, 75 to 79 years), model 3: adjustment for age and school education (Hauptschule, POS/Realschule, Hochschulreife, other/no qualification). For dichotomous variables, the reference category is not shown. Example of interpretation: In male persons with the diagnosis of a myocardial infarction, the odds of being LIFE-Adult participant is 0.50 times as big as the odds of those without a diagnosis of myocardial infarction (model 1)

^a^School qualification: Hauptschule = certificate of primary education, POS/Realschule = certificate of polytechnic secondary school/secondary education, Hochschulreife = technical college or university entrance qualification. ^b^Smoking status refers to cigarette smoking. y = years

After adjustment for differences in the age distribution, physical condition remained associated with study participation only in men (Table 2, model 2). In women, the odds of study participation was also lower among current smokers albeit weaker than in men. Additionally, the odds of being LIFE-Adult participant was higher among married persons in men. The associations of education, employment, and diagnosed diseases with study participation remained directed as in the unadjusted models, although slightly attenuated.

After further adjustment for school education, the above mentioned associations between the individuals’ characteristics and study participation were still present and only slightly weakened (Table 2, model 3).

### Completeness of the data

In LIFE-Adult, the completeness of the data was very high (≥ 98.4%) for variables that had been assessed by interview (see Table 3 for a selection of variables). For these variables, the completeness was lower in short questionnaire participants but above 95%, except for school education. Among those older than 60 years, the completeness was lower in LIFE-Adult than in short questionnaire participants for variables that had been assessed by questionnaires in LIFE-Adult, namely smoking and physical condition. The percentage of available data was lowest among women aged 70 to 79 for questionnaire variables in LIFE-Adult (about 86%) and for all characteristics in short questionnaire participants (mainly about 95%).

**Table 3:** Completeness (%) of selected variables in LIFE-Adult participants and short questionnaire participants by sex and age

	Men				Women			
Age, years	40 to 49	50 to 59	60 to 69	70 to 79	40 to 49	50 to 59	60 to 69	70 to 79
School education								
LIFE-Adult participants	100	100	99.9	99.7	100	99.9	99.9	100
SQ participants	93.8	93.5	93.9	93.9	96.4	95.7	95.1	91.9
Employment								
LIFE-Adult participants	100	99.9	99.6	99.9	100	99.9	99.7	99.9
SQ participants	95.2	95.5	96.5	97.1	96.9	96.9	97	95.5
Cigarette smoking								
LIFE-Adult participants	97.4	95.3	94.0	89.9	97.8	97.2	95.1	86.8
SQ participants	95.2	94.7	95.8	96.8	96.4	96.8	94.6	94.0
Physical condition								
LIFE-Adult participants	97.9	96.4	94.4	87.6	98.5	96.6	91.7	85.5
SQ participants	97.2	96.6	96.8	97.7	97.9	97.5	97.0	95.0
Myocardial infarction								
LIFE-Adult participants	98.7	99.4	98.8	98.6	99.7	99.1	99.6	98.9
SQ participants	95.8	97.5	96.3	96.5	97.7	97.3	96.9	94.8

Completeness is defined as the number of non-missing data divided by the total number of the sample. Sample 2 of LIFE-Adult participants (see Fig. 1) was used. SQ = short questionnaire

### Reasons for nonparticipation

In the raw data, reasons for nonparticipation were distributed as follows: lack of time 21.3%, job-related reasons 2.4%, no interest 12.6%, doubts about the value of the study 3.9%, health reasons 11.7%, moved 0.7%, language reasons 0.9%, other reason 5.7%, multiple answers 13.6%, no information on reasons (including missing data) 27.2%.

After data preparation, six categories of nonparticipation reasons remained. “Lack of time” was the most frequent reason with 30.3%, followed by “no interest” with 19.0% and “health reasons” with 14.3%. The categories “other reason” and multiple answers contained 6.0 and 4.2%, respectively. From 26.2% of the respondents, no reason for nonparticipation was available. Within the “other reasons”, “enough medical care” was mentioned particularly often (in total 2.4%).

**Fig. 3:**
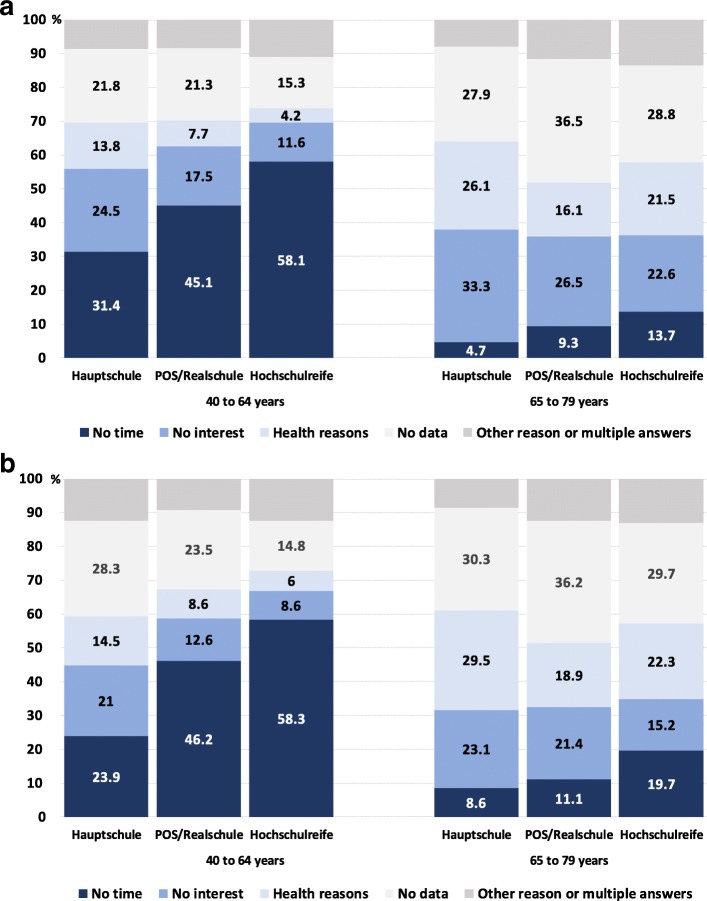
Reasons for nonparticipation according to age and school qualification. **a.** Men. **b.** Women. School qualification: Hauptschule = certificate of primary education, POS/Realschule = certificate of polytechnic secondary school/secondary education, Hochschulreife = technical college or university entrance qualification

“Lack of time” was by far the most common reason among the younger respondents (40 to 64 years) and was reported much more frequently in this group (Fig. 3). In contrast, the older respondents (65 to 79 years) gave “health reasons” much more frequently, as well as “no interest” and no reason for nonparticipation. Respondents with high school education stated time reasons much more frequently and had less missing information (in the younger age group only). In contrast, lower educated persons more often answered with “no interest” and “health reasons”. There was also a tendency of men giving more often “no interest” as the reason for nonparticipation compared to women.

## Discussion

### Key results

LIFE-Adult is a cohort study aimed at providing prevalence and incidence estimates for the Leipzig population, as well as insights into the development of common diseases.

In the study’s main age range from 40 to 79, 31% of the invited persons participated in the baseline examination.

We compared these study participants with both the target population and short questionnaire participants to evaluate the potential for biased study results due to selective participation. Both approaches suggest that participants in LIFE-Adult are less often elderly women and more often married, highly educated, employed, and current nonsmokers. In addition, the data of the short questioning point to LIFE-Adult participants being less often in poor health. The differences between LIFE-Adult participants and the comparison populations were particularly pronounced in education and health variables. Besides, they were partly stronger in men than in women.

Nonparticipation in LIFE-Adult was most often justified with lack of time, lack of interest, and health problems. The reason for nonparticipation strongly depended on age and education of the respondent.

### Strengths and limitations

In contrast to some other countries [5, 13], access to informative data on all potential study participants is very limited in Germany. We used two of the available and particularly meaningful methodological approaches to investigate the potential for selection bias.

First, we compared study participants with the target population by means of census and microcensus data representing a gold standard for the purpose of our investigation. This comparison considers selection factors not only related to the willingness to study participation but also to recruitment procedures [2]. Moreover, representative data meeting high quality standards [19] could be used for that analysis. However, only few relevant characteristics were available from official statistics. In addition, using only summary data, we do not know the statistical precision of the estimates. Furthermore, smoking status was based on voluntary data. However, given the high response to this question (77 to 90%, depending on sex and age strata), the estimated frequencies should be generalizable to the Leipzig population.

Second, we compared study participants with nonparticipants who had filled in a short questionnaire. A broader set of relevant variables could be considered for that. However, questionnaire data were available from only one third of all nonparticipants, which is somewhat lower than in previous studies [8–10, 22, 23]. Consequently, the distributions in short questionnaire participants may not be generalizable to all nonparticipants. In fact, nonparticipants have been characterised as a heterogeneous group [11, 23]. Moreover, we found differences in the completeness of the data between LIFE-Adult and short questionnaire participants that may have affected our comparisons.

Both the sample of the target population and short questionnaire participants differed from LIFE-Adult participants regarding the measurement of study variables (Additional file 1: Table S1). Therefore, the observed deviations of study participants from the comparison populations may partly reflect differences in methodology as well.

Finally, a high percentage of short questionnaire participants did not give the reason for nonparticipation, as reported in other studies [8, 24]. Hence, the distribution and correlates of nonparticipation reasons may not have been validly assessed in this population.

Despite relevant limitations, our findings are plausible, internally consistent, and in line with previous research as discussed below.

### Interpretation of the results

#### Participation in LIFE-Adult

The baseline participation in LIFE-Adult was substantially lower than in previous cohort and cross-sectional studies in Germany and worldwide [1, 6–13, 25–28], with reported median participation of above 70% [1]. This may be mainly due to the steady decline in participation in epidemiologic research over the past about four decades [1, 5]. Less extensive recruitment procedures [13] and certain characteristics of the target population, as presence of higher age groups and urbanity [25, 27], may have contributed to the comparatively low response.

#### Reasons for nonparticipation

Our data on reasons for nonparticipation suggest that time and health constraints, as well as lack of interest contributed to the low participation. They are in line with other epidemiologic studies after which nonparticipation is predominantly justified with lack of time and/or interest [7–9, 22–24, 28, 29]. Health reasons have been frequently given in some studies, too [8, 9, 23, 29]. An accumulation of time reasons in younger persons and health reasons in older persons has also been reported [28, 29]. We additionally found that higher educated persons more often cite time constraints, whereas lower educated persons more frequently mention lack of interest and health problems.

The length of the assessment is an important determinant of participation [17]. Studies requiring a substantial amount of time have lower response rates than studies with lower participant burden [17, 30, 31]. The extent of our baseline assessment may have been an obstacle to participation, especially for persons in the working age and those with a strong professional commitment.

The topic under investigation often influences response most [17]. People are much more interested in participating in a study that explores an issue particularly salient to their lives [30, 31]. The objectives of LIFE-Adult had been broadly formulated so that many of the invited persons might not have seen a personal significance. A diminishing enthusiasm for science in general could be of relevance, too [31]. As our data suggest, a lack of interest in (this kind of) research is of particular concern in subgroups of the population.

Finally, participation in an epidemiologic study can be demanding in many respects [31]. Our complex study design may have meant a great burden, particularly for the elderly who have limited physical resources – but regrettably also the diseases under study.

#### Selective participation in LIFE-Adult

It is widely recognised that not low participation itself but differences between participants and nonparticipants in relevant characteristics threaten the validity of a study [4, 5, 17]. Our investigation suggests that those who participated in LIFE-Adult considerably differ from those not included in the study, particularly in terms of education and health status.

Our results are consistent with previous research that has predominantly shown that participants in epidemiologic studies are more likely to be married, highly educated, and employed in comparison with nonparticipants (e.g., [6–10, 12–15, 28, 32]. The latter two characteristics are related to higher social status. On the one hand, persons with higher social status may be more time constrained. On the other hand, their overrepresentation in epidemiologic studies likely reflects greater health awareness and interest in science [31].

Our findings are also in accord with the observation that nonparticipants in epidemiologic studies more often report poor subjective health [6–8, 10, 12]. Our data further indicate that persons diagnosed with a common disease are less likely to participate in studies like ours. The impact of prevalent diseases on study participation has been investigated with conflicting results. Both no relation between disease status (including cardiovascular diseases, stroke, and diabetes) and response [6, 14] and lower participation rates among diseased persons [10, 15, 26, 28, 33], as well as higher participation associated with disease [11, 23, 32] have been reported. The possible underrepresentation of ill persons in LIFE-Adult may be explained with several mechanisms, including lower health awareness, physical constraints hampering study participation, already high burden by frequent visits to the doctors, and satisfactory medical care (of course, representing a misunderstanding of the study’s aims).

Furthermore, our data are consistent with available evidence after which current smokers are underrepresented among study participants [6–9, 14, 15, 28]. An unhealthy lifestyle is likely to be related to lower identification with the objectives of an epidemiologic study. Also, studies that are perceived to be concerned with socially undesired behaviour may have difficulties to recruit participants who practise such behaviour [31].

Our observation that older people, in particular women, are less likely to be among the study participants is in line with some studies, too (e.g., [8, 10, 13, 22, 23, 29]. Especially elderly women refused to participate because they had to take care for relatives, mostly their husbands [8]. We found that also the response to the study’s questionnaires was lowest among elderly women, as observed in another study [34]. This might partly reflect low familiarity with modern methods of data collection, as a preference for the paper to the computer versions of our questionnaires among elderly women indicates (data not shown).

Our results suggest that selection into the study population may be more pronounced in men than in women, whereas little difference seems to exist between age groups in the range from 40 to 80. Our findings are corroborated by few studies that also observed stronger relations of response to marital status, education, smoking status, and subjective health among males [8, 9, 33], whereas age did not modify these associations [8, 15]. Our observation supports the hypothesis that less health-conscious men are less willing to participate in surveys than their female counterparts [35].

It is often argued that studies with a low response, typically below about 50%, are particularly prone to selection bias [1, 17, 24]. However, studies with substantially higher response than LIFE-Adult, largely between 50 and 75%, mainly reported differences between participants and nonparticipants qualitatively similar to those found in our study as discussed above. The magnitude of these differences was also sizable in various studies (e.g., [8, 10, 13, 28]. In line with these findings, a marked increase in response in a health survey from 37 to 60% brought about by multiple reminders did not eliminate existing differences between participants and nonparticipants [13].

#### Impact of selective participation on study results

Selective participation in epidemiologic studies primarily affects the description of the health status of a population [36–39]. For that purpose, study participants have to be representative of the target population with respect to the characteristics of interest. Therefore, as a consequence of overrepresentation of healthy and health-conscious persons in LIFE-Adult, frequencies of major risk factors and diseases in the Leipzig population will likely be underestimated. Weighting the study data to match the target population distribution for selected socio-demographic features is a common approach to correct for nonresponse in prevalence estimates [2, 22]. The census and microcensus data inform us about the distribution of important socio-demographic characteristics in the Leipzig population, thus enabling us to calculate corresponding weighting factors. However, our regression models suggest that the differences between LIFE-Adult participants and nonparticipants in lifestyle and health variables may be attributed only to a small extent to differences in the distributions of age and education. Thus, weighting prevalence estimates of lifestyle and health characteristics for socio-demographic factors might insufficiently adjust for selection bias in LIFE-Adult.

The validity of analytic-epidemiologic studies is not necessarily impaired by selective participation [36–39]. Estimates of exposure-outcome associations may be biased if selection into the study population depends on both the exposure and the outcome [2, 5]. This situation, also termed differential selection, might particularly affect the internal validity of cross-sectional studies [1]. Evidence for such bias comes from studies that could compare associations among study participants with those in the target or the total nonparticipant population. Among survey participants with low socio-economic status, subjective health was better compared to corresponding census participants [12]. As a result of this differential selection, the survey underestimated the relation of socio-economic status to health. Furthermore, baseline associations between socio-demographic variables and health status partly differed in direction between participants in a cohort study and nonparticipants [27]. We did not examine selection bias at estimates of cross-sectional relations due to the lack of relevant data on the target population and the likelihood of selective participation even in the short questioning. However, a differential selection related to sex as indicated by our findings may bias the effects of sex on health conditions [35].

The validity of longitudinal studies is assumed to be primarily threatened by selective loss to follow-up, whereas selection at baseline is considered rather harmless [30]. There are indications that participation in follow-up examinations follows similar selection patterns as participation at recruitment, particularly with regard to socio-demographic and lifestyle factors [5, 25]. Yet, existing evidence suggests that effects on selected exposure-outcome associations are generally small as differential selection seems to be modest [5, 40]. However, the actual impact of selective participation, both at baseline and at subsequent follow-ups, on the validity of prospective studies has to be further explored [4, 5, 40].

## Conclusions

Our investigation suggests that the comparatively low baseline participation in LIFE-Adult is associated with the typical selection of study participants with higher social status and healthier lifestyle, as well as fewer diagnosed diseases. In particular, education and health status seem to be crucial selection factors. Consequently, primarily frequencies of major risk factors and diseases in the general population will likely be underestimated. Our data support existing evidence that selective participation may be more pronounced in men than in women, which might also distort effect estimates. More informative data on the target population and/or a representative sample of nonparticipants would be necessary to assess the actual selection bias in the study results.

In accord with prior research and the characteristics of LIFE-Adult participants, lack of time and interest as well as health problems frequently deterred invited persons from study participation. Therefore, these issues should be considered in the continuation of LIFE-Adult and in similar studies to raise participation and to minimise selection bias.

## Additional file

Additional file 1: **Table S1.** Definition of the analysis variables in LIFE-Adult participants, the Leipzig population, and short questionnaire participants. (DOCX 19 kb)

## Data Availability

The datasets analysed in the present study are available from the corresponding author on reasonable request.

## Abbreviations

ISCED 97: International Standard Classification of Education 1997
LIFE: Leipzig Research Centre for Civilization Diseases
POS: Polytechnic secondary school
SQ: Short questionnaire
vs.: Versus
