# Supplementary material for: Evaluating selection bias in a population-based cohort study with low baseline participation: the LIFE-Adult-Study
Source: BMC Med Res Methodol. 2019 Jul 1;19:135. doi: 10.1186/s12874-019-0779-8 (PMC6604357; doi:10.1186/s12874-019-0779-8)
Supplement: Supplementary file 1 — Table S1. Definition of the analysis variables in LIFE-Adult participants, the Leipzig population, and short questionnaire participants. (DOCX 19 kb) [file 12874_2019_779_MOESM1_ESM.docx]

**Table S1** Definition of the analysis variables in LIFE-Adult participants, the Leipzig population, and short questionnaire participants

|  | LIFE-Adult participants | Leipzig population | Short questionnaire participants |
| --- | --- | --- | --- |
| **Sex** |  |  |  |
| Data source | Registration office | Census | Registration office |
| Classification for analysis | Male  Female | Male  Female | Male  Female |
|  |  |  |  |
| **Age** |  |  |  |
| Data source | Registration office | Census | Registration office |
| Classification for analysis | 5-year groups | 5-year groups | 5-year groups^1^ |
|  |  |  |  |
| **Marital status** |  |  |  |
| Data source | Interview | Microcensus: Interview | Questionnaire |
| Questions | Which marital status do you have? | Which marital status do you have? | Which marital status do you have? |
| Classification for analysis | Married (living together with spouse, living separated from spouse)  Unmarried (single, divorced, widowed) | Married*  Unmarried (single, divorced*, widowed*)  *also refers to registered partnership | Married*  Unmarried (single, divorced*, widowed*)  *also refers to registered partnership |
|  |  |  |  |
| **Education** |  |  |  |
| Data source | Interview | Microcensus: Interview | Questionnaire |
| Questions | Which highest general school leaving certificate do you have?  Which vocational qualification do you have? | Which highest general school leaving certificate do you have?  Which highest vocational qualification do you have? | Which highest general school leaving certificate do you have? |
| Classification for analysis | Considering school and vocational qualification [S1,S2]:  High education (first stage of tertiary education)  Medium and low education (primary, lower secondary, upper secondary, and post-secondary non-tertiary education) | Considering school and vocational qualification [S1,S2]:  High education (first stage of tertiary education)  Medium and low education (primary, lower secondary, upper secondary, and post-secondary non-tertiary education) |  |
|  |  |  |  |
|  | Considering school qualification:  Hochschulreife (technical college or university entrance qualification)  Polytechnische Oberschule (POS) or Realschule (certificate of polytechnic secondary school or secondary education)  Hauptschule (certificate of primary education)  Other or no qualification |  | Hochschulreife (technical college or university entrance qualification)  Polytechnische Oberschule (POS) or Realschule (certificate of polytechnic secondary school or secondary education)  Hauptschule (certificate of primary education)  Other or no qualification |
|  |  |  |  |
| **Employment** |  |  |  |
| Data source | Interview | Microcensus: Interview | Questionnaire |
| Questions | Are you currently gainfully employed? | Employment within the last week: Which group do you belong to? | Are you currently gainfully employed? |
| Classification for analysis | Employed (having a paid job regardless of duration)  Unemployed or inactive persons | Employed (having a paid job regardless of duration)  Unemployed or inactive persons | Employed (not further defined)  Unemployed or inactive persons |
|  |  |  |  |
| **Smoking status** |  |  |  |
| Data source | Interview, questionnaire | Microcensus: Interview | Questionnaire |
| Questions | Have you smoked regularly in your life for more than 6 months? If “Yes”: Do you smoke currently – if only occasionally?  Additional questions related to the amount of consumption of different tobacco products | Do you smoke currently (if only occasionally)? | Do you smoke cigarettes currently? If “No”, did you smoke in the past? |
| Classification for analysis | Current smoker  Current nonsmoker (former or never smoker) | Current smoker  Current nonsmoker (former or never smoker) |  |
|  |  |  |  |
|  | Considering cigarette smoking:  Current cigarette smoker  Former cigarette smoker  Never cigarette smoker |  | Current cigarette smoker  Former cigarette smoker  Never cigarette smoker |
|  |  |  |  |
| **Physical condition** |  | *Not available* |  |
| Data source | Questionnaire [S3] |  | Questionnaire |
| Questions | How did you feel within the last seven days? Physically, I am in a poor condition: scale from 1 (yes, that is true) to 5 (no, that is not true) |  | How would you describe your current physical condition? |
| Classification for analysis | Poor (1)  Not poor (2 to 5) |  | Poor  Not poor (less well, good, very good) |
|  |  |  |  |
| **Disease diagnoses** |  | *Not available* |  |
| Data source | Interview |  | Questionnaire |
| Questions | Have you ever been diagnosed with a myocardial infarction/stroke/diabetes/cancer by a doctor? |  | Have you ever been diagnosed with a myocardial infarction/stroke/diabetes/cancer by a doctor? |
| Classification for analysis | Yes  No |  | Yes  No |

Answer categories were given for all questions, except for the amount of tobacco consumption. To keep the table readable, these categories are not shown.

^1^For those who had been unwilling to participate in LIFE-Adult, information on age were exclusively available in 5-year groups for privacy reasons.

S1. International Standard Classification of Education ISCED 1997. <http://www.unesco.org/education/information/nfsunesco/doc/isced_1997.htm> Accessed 18 July 2018.

S2. Statistische Ämter des Bundes und der Länder, editors. Internationale Bildungsindikatoren im Ländervergleich. Wiesbaden 2006. <https://www.destatis.de/GPStatistik/receive/DEHeft_heft_00010329> Accessed 18 July 2018.

S3. Smets EM, Garssen B, Bonke B, de Haes JC. The Multidimensional Fatigue Inventory (MFI) psychometric qualities of an instrument to assess fatigue. J Psychosom Res. 1995;39:315–25.
